# Case report: Long-term progression-free survival in advanced ovarian cancer treated with apatinib in first-line maintenance treatment

**DOI:** 10.3389/fonc.2024.1481251

**Published:** 2024-10-28

**Authors:** Ning Zan, Xuan Zhang, Danfei Yu, Juan Liu, Zhiyu Lin, Yanlin Zhu

**Affiliations:** ^1^ Department of Oncology And Hematology, People’s Hospital of Leshan, Leshan, China; ^2^ Pulmonary and Critical Care Medicine, People’s Hospital of Leshan, Leshan, China

**Keywords:** ovarian cancer, maintain therapy, angiogenic, apatinib, gynecology, case report

## Abstract

Ovarian cancer is one of the most common gynecological malignancies. The current first-line treatment strategies for advanced ovarian cancer include surgery, chemotherapy, and maintenance therapy. Bevacizumab and poly (ADP-ribose) polymerase inhibitors (PARPi) are primary maintenance treatments for advanced ovarian cancer. Previously, many patients declined these therapies before medicare coverage because of high costs. Bevacizumab and apatinib are anti-tumor angiogenic agents. In this case study, we describe a patient with advanced ovarian cancer who underwent neoadjuvant chemotherapy, interval debulking surgery, and adjuvant chemotherapy. She declined bevacizumab and PARPi maintenance therapy owing to the prohibitive expenses. The patient was administered off-label apatinib and achieved a progression-free survival of 54 months. Thus, apatinib may offer substantial therapeutic value as a first-line maintenance therapy in advanced ovarian cancer.

## 1 Introduction

Ovarian cancer is a serious threat to women’s health. Even though its incidence is lower than that of gynecological cancers of the cervix and uterus. It is the second most common gynecological cancer with the highest mortality rate (1). Most patients are diagnosed at an advanced stage owing to a lack of symptoms and other reasons (2). Therefore, patients with ovarian cancer have a poor prognosis. Prior to the introduction of targeted therapies, the standard treatment for ovarian cancer primarily involved surgery accompanied by platinum-based chemotherapy. The integration of poly (ADP-ribose) polymerase inhibitors (PARPi) and bevacizumab as first-line maintenance therapies has revolutionized the treatment paradigm of ovarian cancer, significantly prolonging the progression-free survival (PFS) and overall survival (OS) in patients with advanced stages of the disease (3–5). Despite their benefits, many patients declined the use of PARPi and bevacizumab owing to their high costs, which were not being covered by the national health insurance. Both apatinib and bevacizumab serve as anti-tumor angiogenic agents, with apatinib being efficacious as either part of combination regimens or as a monotherapy in treating recurrent ovarian cancer (6, 7). Further, apatinib costs lesser than bevacizumab and PARPi. However, the use of apatinib as first-line maintenance therapy in ovarian cancer has not been reported to date. This report documents a case of prolonged PFS achieved in a patient with stage IVb ovarian cancer using apatinib as a first-line maintenance therapy.

## 2 Case description

**Figure 1:**
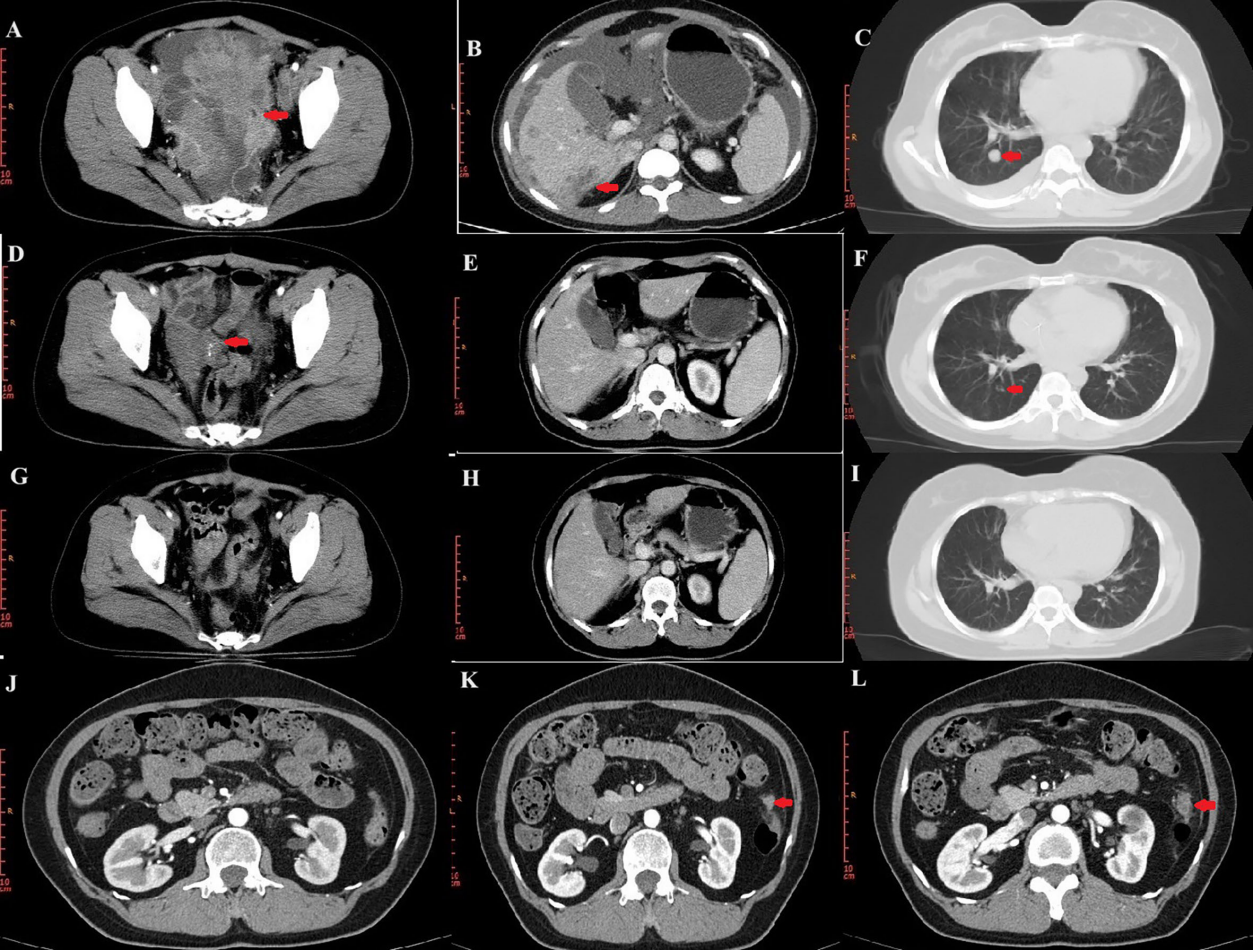
CT images during treatment. **(A–C)** CT image of the lesion at initial treatment. Pelvic lesions **(A)**. Liver lesions **(B)**. Lung lesions **(C)**. **(D–F)** CT images after three cycles of chemotherapy. **(G–I)** CT images at the end of chemotherapy. **(J)** No tumor recurrence on CT on April 19, 2023. **(K, L)** Lesions of peritoneum on CT.

**Figure 2:**
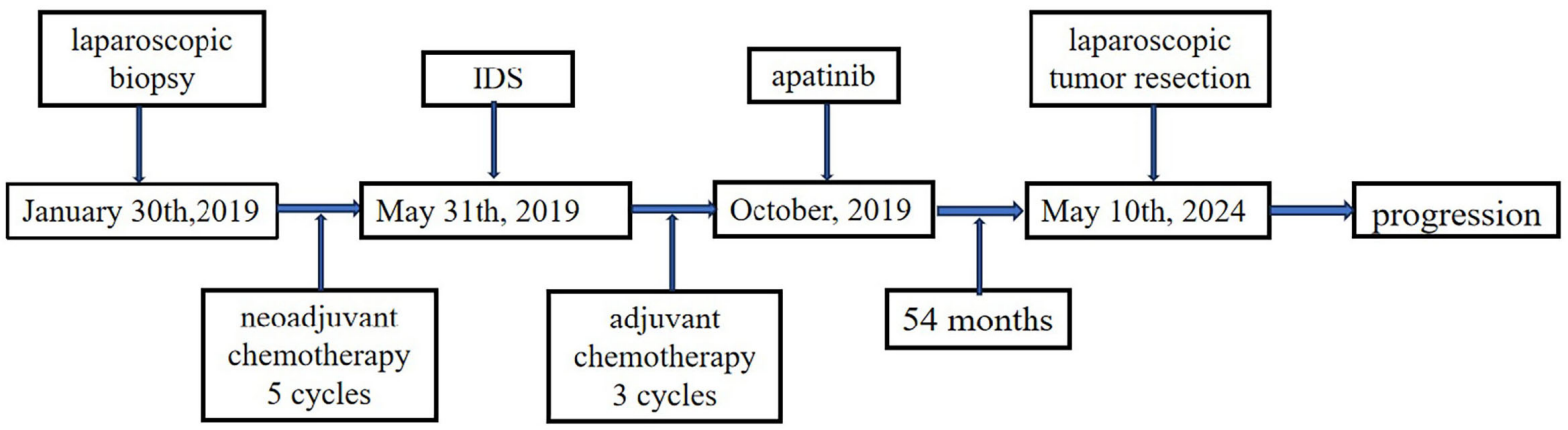
Timeline of the patient’s treatment. PARPi, Poly (ADP-ribose) polymerase inhibitors; PFS, Progression-free survival; OS, Overall survival; CT, Computed tomography; CA125, Carbohydrate antigen 125; AUC, Area under the curve; IDS, Interval debulking surgery; CTCAE, Common Terminology Criteria for Adverse Events; VEGF, Vascular endothelial growth factor; VEGFR, VEGF receptor.

In November 2018, a 44-year-old female was admitted to a local hospital for abdominal pain. The medication she received during this period is not known. Symptomatic treatment provided no obvious relief. Previously, the patient had undergone a hysterectomy for uterine fibroids at the age of 39 years. She had no family history of cancer. In January 2019, computed tomography (CT) scans revealed no normal uterine and ovarian tissues. Multiple masses were observed on both sides of the pelvis, with the largest cross-section measuring approximately 7.6×6.2 cm (**Figure 1A**). Multiple masses were observed in the peritoneum and omentum. Moreover, multiple metastatic lesions were observed in the liver and lungs (**Figures 1B, C**). Enlargement of the abdominal, retroperitoneal, and cardiophrenic angle lymph nodes was suggestive of metastasis. The level of carbohydrate antigen 125 (CA125) was elevated at 486.0 U/ml. On January 30, 2019, the patient underwent a laparoscopic biopsy of the peritoneal lesions. Histopathological and immunohistochemical analyses confirmed high-grade serous adenocarcinoma of ovarian origin. Immunohistochemical staining results were as follows: CK7(+++), Vim(-), P53(++), P16(+++), WT-1(+++), CA125(+++), CA199(-), CK5/6(-), P63(-), CK7(+), CK20(-), Villin(-), ER (partially +), PR(-), PAX-8 (partially +), Ki67 (+; approximately 60%). The patient was diagnosed with stage IVb high-grade serous adenocarcinoma of the ovary. On February 1, 2019, the patient commenced treatment with paclitaxel (175 mg/m2, intravenously, day 1) and carboplatin (AUC 5, intravenously, day 1), administered every three weeks for a total of three cycles. On April 2, 2019, a CT scan revealed a significant reduction in all lesions (**Figures 1D–F**). However, achieving satisfactory tumor resection based on the Suidan score remains challenging. The patient continued with the original chemotherapy regimen for two cycles before undergoing interval debulking surgery (IDS) on May 31, 2019. This procedure included the resection of bilateral ovarian tissues, fallopian tubes, peritoneum, omentum, appendix, and enlarged lymph nodes. Histopathological and immunohistochemical analyses confirmed a high-grade serous adenocarcinoma of the right ovary. The left ovarian inclusion cyst was calcified and contained foam cell aggregations. Notably, peritoneal and omental tissues exhibited fibrous hyperplasia with numerous foam cell aggregates. No tumor cells were present in the fallopian tubes, appendix, or lymph nodes bilaterally. The immunohistochemical staining yielded positive results for Vim (nucleus+), CA125(+), CK7(+), P53(+), P16(+), ER(+), and Ki67 (approximately 25%), with negative results for PR(-) and CK20(-). Postoperatively, the patient declined bevacizumab because of its high cost and instead completed three more cycles of the initial chemotherapy regimen. Subsequently, a CT scan on September 10, 2019, revealed the resolution of tumor metastases in the abdomen, pelvis, lungs, and liver (**Figures 1G–I**). The patient declined genetic testing and PARPi maintenance therapy owing to the high costs. She consented to receive off-label apatinib (750 mg, oral, QD) starting in October 2019. Subsequently, she developed palmar-plantar erythrodysesthesia syndrome (Common Terminology Criteria for Adverse Events [CTCAE] grade 3) and requested a dose adjustment. After the daily dose was reduced to 500 mg, her symptoms improved. The patient remained stable with regular follow-ups, and a CT scan on April 19, 2023, showed no signs of tumor recurrence (**Figure 1J**). Computed tomography (CT) revealed new lesions on the left side of the peritoneum and omentum in April 2024 (**Figures 1K, L**). The patient underwent laparoscopic tumor resection for peritoneal and omental diseases on May 10, 2024. Histopathological and immunohistochemical analyses indicated a high-grade serous adenocarcinoma of the female reproductive system. Immunohistochemical staining results were as follows: CK(AE1/AE3)(+), Syn(-), Pax-8(+), S100(-), SALL4(-), Ki67(+; about 80%). Recurrence of ovarian cancer was determined based on patient history and pathology findings. The patient achieved a PFS of 54 months following first-line maintenance therapy with apatinib. The timeline of the patient’s treatment is shown in **Figure 2**.

## 3 Discussion

Advanced ovarian cancer has a poor prognosis. In recent years, bevacizumab and PARPi have been shown to improve the prognosis of patients with advanced ovarian cancer (3–5). Tumor growth requires blood vessels to provide oxygen and nutrients. Furthermore, the vascular endothelial growth factor (VEGF) plays an important role in the process of angiogenesis (8). Notably, VEGF expression is higher in ovarian tumor tissues than in normal and benign ovarian tissues (9). There are two different clinical treatment strategies for VEGF. Bevacizumab, an anti-VEGF monoclonal antibody, inhibits the proangiogenic effect of VEGF. Another clinical treatment strategy targeting VEGF is inhibiting the function of the VEGF receptor (VEGFR), using apatinib for example. VEGF, especially VEGF-A, has been identified as a key factor in tumor angiogenesis. Bevacizumab inhibits the binding of VEGF-A to VEGFR tyrosine kinases (VEGFR1-3), inhibits tumor vascular growth, promotes tumor vascular normalization, and causes tumor cell death (10). Moreover, VEGFR2 is the main signaling pathway of VEGFR in vascular endothelial cells (11). Apatinib is a small oral VEGFR-2 tyrosine kinase inhibitor molecule that inhibits tumor angiogenesis by blocking downstream signaling (7).

Apatinib, alone or in combination with chemotherapy, has shown favorable antitumor effects and manageable safety in patients with platinum-resistant or platinum-refractory ovarian cancer (12, 13). Furthermore, it improves the overall survival (OS) in patients receiving third-line treatment for gastric cancer or gastroesophageal junction carcinoma (14). Apatinib plus camrelizumab has a survival benefit as a first-line therapy for unresectable hepatocellular carcinoma (15). Notably, patients with advanced ovarian cancer also benefit from first-line maintenance therapy with bevacizumab (4, 16). However, there have been no clinical studies on apatinib as a first-line maintenance therapy for ovarian cancer.

Till date, no studies have compared the efficacy of apatinib versus bevacizumab in ovarian cancer maintenance therapy. However, animal studies have shown that apatinib has stronger antitumor activity than bevacizumab in transgenic zebrafish embryo and human lung cancer xenotransplantation models (17). In advanced gastric cancer, apatinib is more effective than bevacizumab (18). The study of real-world drug treatment models of novel targeted drugs showed that the total drug costs and per capita drug cost of bevacizumab or PARPi were higher than those of apatinib in Chinese patients with gynecological cancer from 2017 to 2021 (19). The price of bevacizumab was significantly higher than apatinib before the national health insurance coverage and the price of bevacizumab is still higher than apatinib after the national health insurance coverage in China (20).

In the current case, advanced ovarian cancer was suspected at the initial diagnosis. The patient received neoadjuvant chemotherapy, interval debulking surgery, and adjuvant chemotherapy. The patient declined bevacizumab and PARPi maintenance therapy owing to their high costs. Consequently, the patient opted for the less costly off-label apatinib maintenance therapy. The patient attained a long-term PFS of up to 54 months. This outcome holds potential therapeutic value for first-line maintenance in advanced ovarian cancer. However, some patients may experience adverse events. Since this is a single case, which is a limitation of this study, it is not known whether this treatment model will be applicable to other patients with advanced ovarian. Further studies and clinical trials are necessary to verify the effectiveness of this strategy.

## Data Availability

The original contributions presented in the study are included in the article/supplementary material. Further inquiries can be directed to the corresponding author.
